# Impact of operating room waste in a high-volume institution and strategies for reduction: results from the CARING NATURE project

**DOI:** 10.1093/bjs/znaf027

**Published:** 2025-02-17

**Authors:** Laura Lorenzon, Sabina Magalini, Laura Antolino, Giulia De Rubeis, Lorenzo Ferri, Cristina Galati, Gloria Santoro, Pasquale Mari, Benedetto Bresa, Daniele Gui, Caring Nature Working Group, Paola Aceto, Rossana Addei, Sergio Alfieri, Salvatore Agnes, Andrea Cambieri, Claudio Coco, Domenico D'Ugo, Michele Di Donato, Felice Giuliante, Carlo Licorni, Ersilia Luca, Stefano Margaritora, Fabio Pacelli, Marco Racioppi, Marco Raffaelli, Giovanni Scambia, Luigi Sofo, Liliana Sollazzi, Silvia Tirabassi, Yamume Tshomba

**Affiliations:** Fondazione Policlinico Universitario Agostino Gemelli IRCCS, Rome, Italy; Catholic University of the Sacred Hearth, Rome, Italy; Fondazione Policlinico Universitario Agostino Gemelli IRCCS, Rome, Italy; Unit of Oncologic and General Surgery, Belcolle District Hospital, Viterbo, Italy; Fondazione Policlinico Universitario Agostino Gemelli IRCCS, Rome, Italy; Fondazione Policlinico Universitario Agostino Gemelli IRCCS, Rome, Italy; Fondazione Policlinico Universitario Agostino Gemelli IRCCS, Rome, Italy; Fondazione Policlinico Universitario Agostino Gemelli IRCCS, Rome, Italy; Catholic University of the Sacred Hearth, Rome, Italy; Fondazione Policlinico Universitario Agostino Gemelli IRCCS, Rome, Italy; Catholic University of the Sacred Hearth, Rome, Italy; Fondazione Policlinico Universitario Agostino Gemelli IRCCS, Rome, Italy

Surgery has a significant environmental impact. Each operating room can produce up to 2300 kg of waste annually^1^ and operating rooms can consume six times more energy than other hospital departments^2^. Anaesthetic gases and energy consumption are the largest sources of greenhouse gas emissions. This is in addition to various waste by-products, including biological, chemical, and non-hazardous waste. Up to 90% of operating room waste is misclassified hazardous by default and incinerated, generating toxic by-products, complicating recycling efforts, and increasing costs^3–4^. There has been a recent focus on the carbon footprint of surgery demonstrated by two-thirds of the manuscripts in this arena having been published in the last 2 years (*Supplementary Materials*).

This two-week audit aimed to measure operating room waste produced across various specialties in a high-volume Italian institution and to identify areas for interventions (*Supplementary Materials*). Fondazione Policlinico Universitario A. Gemelli IRCCS is the largest Italian Research University Hospital with more than 1600 beds and 52 operating rooms (*Supplementary Materials*). The Institution performed more than 91 000 surgical procedures in 2023. The elective operating room suite is composed of 12 operating rooms (*Fig. 1*). *Figure 1*, *Supplementary Methods*, and *Fig. S1* illustrate the organization of the ward and how the surgical waste is managed and disposed of in the operating room using cardboard and polyethylene bins.

**Fig. 1:**
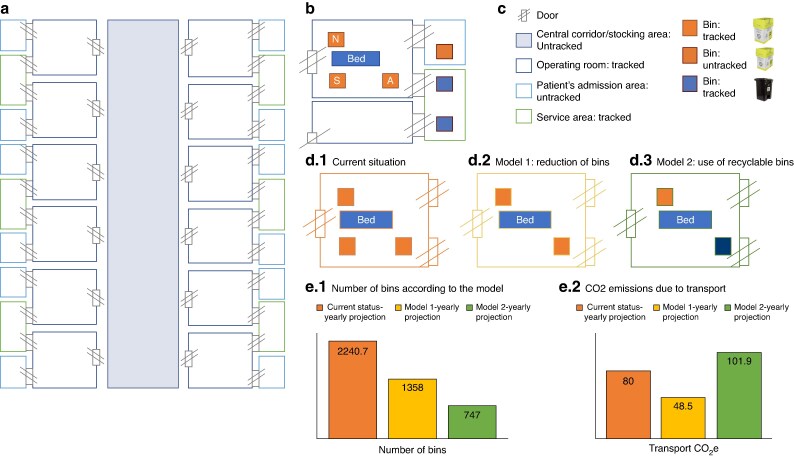
**a** Schematic floorplan of the operating room area. **b** Detail of the bins usually allocated in the operating room (N: cardboard bin for nurses; S: cardboard bin for surgeons; A: cardboard bin for anaesthesiologists). **c** Legend and area tracked/untracked. **d.1** Current situation; **d.2** Model 1: reduction of cardboard bins per operating room; **d.3** Model 2: reduction of bins including the use of recyclable units. **e.1** Total number of bins according to the model (based on the estimation of annual number of colorectal procedures); **e.2** emissions due to transportation according to the model (based on the estimation of the annual number of colorectal procedures)

The study audited surgical procedures performed in the elective operating room suite in June 2024; exclusion criteria were transplant, private practice procedures, and cardiac surgery, neurosurgery, deliveries, private practices, transplants, and C-sections. Emergency and re-allocated procedures performed in the operating rooms were not excluded but registered accordingly. The data captured in the audit are reported in *Supplementary Materials* and *Fig. S2*.

The primary outcome was the amount of waste produced in the operating room expressed by the weight in kilograms and the number of cardboard bins at the end of surgical procedures. Secondary outcomes included: the correlation with surgical activities and personnel involved, the estimation of the annual waste produced by sub-specialty, and the estimation of the emissions (CO_2_ emissions, CO_2_e)^2,5–7^, and costs. Finally, two models for implementation, including reduction of cardboard and sterilization of recyclable polyethylene bins^8^, were also tested (see *Supplementary Materials*). Correlations between surgical and anaesthesiologic practices/variables and the outcomes of interest were tested using univariable analyses and a generalised linear regression model. Secondly, variables were investigated for internal correlations (collinearity). Finally, a structural equation modelling (SEM)^9^ analysis was conducted to investigate causal effects (*Supplementary Materials*).

During audit, 364 surgical procedures were tracked. The number of procedures per day ranged from 30 to 43, with approximately 10% being unplanned. A total of 1033 cardboard bins were used, containing about 2422 kg of waste. The weighted mean waste per procedure was 2.1 kg, with around 7% of the bins weighing 1 kg or less (*Tables S1–S4*). Almost 80% of procedures utilized reusable linen drapes and gowns, although 64% involved personnel using mixed scrubbing methods.

Nearly 40% of procedures were laparoscopic or thoracoscopic, with a median procedure time of 100 min and operating room occupancy lasting an average of 153 min. Clean procedures accounted for about 60%, followed by clean-contaminated in 35%. The median waste produced per minimally invasive procedure was 6.2 kg, with a median of three cardboard bins used (*Tables S1–S4*).

Univariable analyses found that use of non-woven fabric drapes and gowns (*P* < 0.001), the increased use of disposable devices (*P* < 0.001), and converted surgery (*P* < 0.001) and longer procedures (*P* < 0.001) were correlated with an increased median output of waste. The same was reported for increased blood loss and discarded fluids (*P* < 0.001) and the number of personnel (surgeons and nurses, *P* < 0.001) involved in the procedures. The sub-specialty was documented as impacting surgical waste (*P* < 0.001). Also, contaminated procedures (*P* < 0.001), those requiring multiorgan resection (*P* < 0.001), and emergency or reallocated procedures (*P* 0.140) were correlated with greater production of waste. The multivariable analysis found that thoracic surgery (OR 5.3; 95% c.i.: 1.2 to 24.3) and those classified as other sub-specialties (OR 6.1; 95% c.i.: 1.2 to 30.4) had the greatest impact on waste. Clean-contaminated procedures (OR 3.1; 95% c.i.: 1.4 to 7.1), and the number of nurses (OR 1.7; 95% c.i.: 1.03 to 2.9) also increased waste production (*Tables S5–S8*). SEM analysis identified no definitive causality relationships among variables (*Tables S9–S13* and *Fig. S3*). The total waste recorded led to an estimation of emissions of approximately 6714.8 kg CO_2_e from incineration and transportation.

Implementation models based on colorectal procedures suggested a potential for reduction in waste generation. By reducing the average number of cardboard units from 3.3 to 2 per procedure, emissions could decrease by 39.4%. A secondary model, proposing the use of one cardboard and one polyethylene unit (recyclable up to 10 times) per procedure, would decrease travel-related emissions by 66.7%, but the additional emissions from sterilization cycles could negate its effectiveness (*Fig. 1*).

This study provides a comprehensive evaluation of surgical practices related to operating room waste, identifying opportunities to reduce waste. Although some issues, such as multiple organ resections and lengthy procedures, are challenging to address, other sub-specialties could benefit from targeted interventions. The findings highlight significant waste correlation with operating room practices, echoing previous research showing that reusable linens could reduce waste by over 70%^10^. Additionally, reducing the number of cardboard bins in the operating room could impact transportation emissions, as many bins weigh less than 1 kg. This study’s strength lies in its extensive approach across various surgical specialties at a major Italian university hospital. However, limitations include the single-centre focus and exclusion of certain specialties that may affect generalizability (see *Supplementary Materials* for other insights).

This study underscores the importance of integrating sustainable practices into operating room management, requiring time, personnel education, and overcoming cultural barriers. The emissions and waste generated from surgical practices confirm the need for healthcare systems to adopt more sustainable approaches as surgical activity rises globally. Further research should enhance the tailoring of interventions to meet ecological targets.

## Collaborators

Paola Aceto MD, Rossana Addei BSN, Sergio Alfieri MD, Salvatore Agnes MD, Andrea Cambieri MD, Claudio Coco MD, Domenico D'Ugo MD, Michele Di Donato MD, Felice Giuliante MD, Carlo Licorni BSN, Ersilia Luca MD, Stefano Margaritora MD, Fabio Pacelli MD, Marco Racioppi MD, Marco Raffaelli MD, Giovanni Scambia MD, Luigi Sofo MD, Liliana Sollazzi MD, Silvia Tirabassi BSN, Yamume Tshomba MD, Fondazione Policlinico Universitario Agostino Gemelli IRCCS, Rome, Italy.

## Supplementary Material

znaf027_Supplementary_Data

## Data Availability

Research data supporting this publication are available from the corresponding author upon reasonable request.
