# Supplementary material for: Impact of operating room waste in a high-volume institution and strategies for reduction: results from the CARING NATURE project
Source: Br J Surg. 2025 Feb 17;112(2):znaf027. doi: 10.1093/bjs/znaf027 (PMC11831365; doi:10.1093/bjs/znaf027)
Supplement: znaf027_Supplementary_Data [file znaf027_supplementary_data.docx]

**The impact of operating room waste in a high-volume Institution and strategies for reduction: results from the CARING NATURE project**

Laura Lorenzon MD PhD^1^, Sabina Magalini MD^2^, Laura Antolino MD PhD^1,3^, Giulia De Rubeis MD^1^, Lorenzo Ferri MD^1^, Cristina Galati CanMed^1^, Gloria Santoro PhD^1^, Pasquale Mari DEng^2^, Benedetto Bresa DEc^1^, Daniele Gui MD^2^ and CARING NATURE WORGING GROUP^1^*

^1^ Fondazione Policlinico Universitario Agostino Gemelli IRCCS, Rome, Italy

^2^ Catholic University of the Sacred Hearth, Rome, Italy

^3^ Unit of Oncologic and General Surgery, Belcolle District Hospital, Viterbo, Italy

**CARING NATURE WORKING GROUP** (in alphabetical order, all to be indexed in PubMed and Scopus): Paola Aceto MD, Rossana Addei BSN, Sergio Alfieri MD, Salvatore Agnes MD, Andrea Cambieri MD, Claudio Coco MD, Domenico D'Ugo MD, Michele Di Donato MD, Felice Giuliante MD, Carlo Licorni BSN, Ersilia Luca MD, Stefano Margaritora MD, Fabio Pacelli MD, Marco Racioppi MD, Marco Raffaelli MD, Giovanni Scambia MD, Luigi Sofo MD, Liliana Sollazzi MD, Silvia Tirabassi BSN, Yamume Tshomba MD.

**Corresponding author.** Laura Lorenzon, Fondazione Policlinico Universitario A. Gemelli, Catholic University, Largo Francesco Vito 1, 00168 Rome, Italy. email:[laura.lorenzon@policlinicogemelli.it](mailto:laura.lorenzon@policlinicogemelli.it); phone number: +39-0630154974; fax +39-0630158015; **ORCID ID** orcid.org/0000-0001-6736-0383; **Twitter** @LauraLorenzonMD

**Funding source**. The project has received funding from the European Union’s Horizon Europe research and innovation program (HORIZON-HLTH-2023-CARE-04-03: Environmentally sustainable and climate neutral health and care systems) under the Grant Agreement No. 101101322.

**Supplementary Materials - Index**

| **Type - Supplementary Methods** |  |
| --- | --- |
| Pubmed search - literature background | *pag. 2* |
| Design | *pag. 2* |
| Setting: OR floorplan and elective surgical activities | *pag. 2* |
| Surgical waste management in the OR and its disposal | *pag. 3* |
| Study criteria and data collection | *pag. 3* |
| Outcomes of interest | *pag. 4* |
| Statistics | *pag. 4* |
| Disposables and devices | *pag. 5* |
| **Type - Supplementary Results: Figures and Tables** |  |
| Supplementary Table 1. Details of the waste produced in the OR during the audit | *pag. 6* |
| Supplementary Table 2. Data collected during the audit | *pag. 8* |
| Supplementary Table 3. Polyethylene bins breakthrough during the audit | *pag. 10* |
| Supplementary Table 4. Re-allocated/unplanned procedures | *pag. 11* |
| Supplementary Table 5. Univariable analysis for the outcomes of interest: median waste/procedure  and median number of bins/procedure | *pag. 12* |
| Supplementary Table 6. Univariable analysis for the outcome of interest median net waste/procedure | *pag. 14* |
| Supplementary Table 7. Univariable analysis for the outcome of interest median weighted mean waste/procedure | *pag. 16* |
| Supplementary Table 8. Generalised Linear Regression Models | *pag. 18* |
| Supplementary Table 9. Correlations among variables | *pag.20* |
| Supplementary Table 10. Factor analysis | *pag. 21* |
| Supplementary Table 11. Structural equation model (SEM) | *pag. 22* |
| Supplementary Table 12. SEM Performance | *pag. 23* |
| Supplementary Table 13. Confirmatory Factor Analysis of SEM | *pag. 23* |
| Supplementary Figure 1. Map of the Institution | *pag. 24* |
| Supplementary Figure 2. Data captured in the audit | *pag. 24* |
| Supplementary Figure 3. Map of the direct and indirect effects | *pag. 25* |
| **Type - Supplementary Data Interpretation and Limitations** | *pag. 26* |
| **Type - Supplementary References** | *pag. 28* |

**Supplementary Methods**

***Pubmed Search.*** Pubmed search for the query "carbon footprint in surgery" provided 287 manuscripts; 2024: 101 manuscripts; 2023: 100 manuscripts; 2022: 68 manuscripts; 2021: 29 manuscripts; 2020 13 manuscripts; 2019-2002: 24 manuscripts.

*Source:* https://pubmed.ncbi.nlm.nih.gov/?term=carbon+footprint+surgery&sort=pubdate last accessed 22^nd^ September 2024.

***Design****.* This is a no-profit, prospective, spontaneous, pilot cross-sectional study, designed as a 2-week audit, developed within the EU-funded CARING NATURE project. The audit was designed in compliance with the SMART criteria (specific, measurable, agreed, relevant, and theoretically sound, see below). The project has received funding from the European Union’s Horizon Europe research and innovation program (HORIZON-HLTH-2023-CARE-04-03: Environmentally sustainable and climate-neutral health and care systems) under Grant Agreement No. 101101322.

| **SPECIFIC** | **MEASURABLE** | **AGREED** | **RELEVANT** | **THEORETICALLY SOUND** |
| --- | --- | --- | --- | --- |
| Waste output in the Operating Room in a single center high-volume Italian Institution | Waste (kg of waste and number of bins) | Agreed with the Health Management Department of the Institution | Relevant for the Institutional Carbon Footprint. Relevant in scientific literature: 2/3 of the scientific literature published during the last 2 years | The protocol has been developed within an EU-funded project.  Several surgical researches used a 2-week audit design (see: *BMJ Open 2014;4:e006239. doi: 10.1136/bmjopen-2014-006239*  and *BMJ Open 2017;7:e012150. doi: 10.1136/bmjopen-2016-012150*) |

***Setting: OR floorplan and elective surgical activities.*** Fondazione Policlinico Universitario A. Gemelli IRCCS, Catholic University of the Sacred Heart, is located in Rome, Italy, and it is the largest Italian Research University Hospital with more than 1600 beds and 52 operating rooms (ORs) equipped with the latest technological standards, including robotics and the 4K laparoscopy image recording system, see below. The Institution is also a Comprehensive Cancer Centre and provided more than 91.000 surgical procedures in 2023.

The elective OR ward of the Institution is composed of 12 ORs, Figure 1. In brief, there are 6 identical ORs in 2 rows divided by a common central corridor used for storage and supplies.

Each OR has a separate admission area for the patient (induction and scrubbing for OR personnel) and another service area in common with the adjacent room. The ward is also provided with a common recovery area, offices, and services for personnel, whereas the surgical instruments are sterilized in another hospital wing.

The ward is operating for elective procedures Monday-Friday 7.30-20.00 and Saturday 7.30-18.00. Procedures for patients admitted to the Emergency Room are performed in a separate hospital wing, however, rarely, emergency procedures for patients already admitted in the surgical Department are performed in the elective OR ward, re-allocating other elective procedures if required.

The ward is dedicated to the following sub-specialties: colorectal- ileal resections for benign and malignant disease -CRC, liver-hepatobiliary-and pancreatic -HPB, upper-gastrointestinal -UGI-, hernias and abdominal wall procedures, thoracic, vascular, urologic, transplants (liver and kidney), endocrine, and gynaecologic surgeries. One OR of the ward is randomly assigned to private practice each day.

Sub-specialty allocation in the ORs is managed by the Health Management Department and is based on the volume of surgeries performed at the institution each year. In brief, all sub-specialties are assigned to one of the ORs, balancing their allocation to different specialties each month.

All ORs are equipped for laparoscopic/thoracoscopic procedures, and there are 5 robotics systems: two da Vinci Xi (Intuitive Surgical Inc. Sunnyvale - California, USA), two Hugo^TM^ RAS system (Medtronic plc., Dublin, Ireland) and one Versius (CMR Surgical, Cambridge, United Kingdom): two of these systems are for urology and gynaecological procedures, whereas the others are for other sub-specialties based on a monthly rotation. Two ORs of the ward are also equipped for procedures requiring the use of XRs.

Daily coordination is provided by the chief nurse and chief anaesthesiologists aiming to facilitate surgical activities and re-allocate elective procedures (in a different OR of the same ward) if one particular sub-specialty is running late and another has a shorter list of surgical procedures. To the same extent, in the case of transplants, one of the OR lists is randomly cancelled and procedures are re-allocated to the other ORs of the ward based on clinical priorities by the coordination team.

For all of these reasons, and to avoid any fluctuations, a 2-week audit was foreseen as necessary to have a proper estimation of surgical waste produced over time.

***Surgical waste management in the OR and its disposal.*** Figure 1 also illustrates the allocation of waste bins inside and outside each OR.

In brief, and by general rule, each OR has three 60L yellow cardboard bins (one for the anaesthesiologist, one for the scrub nurse, and one for the surgeons) and two collectors for fluids - canisters- (one for the anaesthesiologist and one for the surgeon) that are closed and disposed at the end of every procedure. Another 60L yellow cardboard unit is placed in the patient's admission area, whereas two 60L polyethylene bins where OR personnel discharge larger waste items are placed in the service area shared by two adjacent ORs: these three bins are closed and disposed of at the end of the daily surgical activities. Of note, in the service area is also allocated a device to scan all cardboard bins for the pharmaceutical tags for cost allocations of disposable devices before the final discarding.

Finally, the central corridor is dedicated to the stock for general supplies including drapes, sutures, drains, and disposables of routine use. Here, segregation and waste sorting for plastic and paper are usually practiced, whereas segregation is not performed inside the ORs.

Supply and disposal of the medical bins are provided by an out-sourcing company, but they are supervised and monitored by the Health Management Department of the Institution. All bins are temporarily allocated in a landfill within the Hospital area, Supplementary Figure 1, weighted altogether along with others from different wards and Departments, and then incinerated elsewhere. The transportation is arranged for trucks and trailers when they include altogether 1200 cardboard and polyethylene bins, independently from their weight.

***Study criteria and data collection***. The study included the surgical procedures performed in the elective OR ward over two weeks (Monday-Friday) in June 2024, independently from the sub-specialty.

Of note, emergency and re-allocated procedures performed in the elective ORs were not excluded but registered accordingly, see Supplementary Table 4. Exclusion criteria were transplant, private practice procedures, and those that included: cardiac surgery, neurosurgery, deliveries, and C-sections. Saturday list was not recorded since it usually includes minor procedures.

Data captured in the audit is represented in Supplementary Figure 2. Data collected included: day of the audit; OR identification number; number of cardboard bins in the OR in each procedure (overall and number of cardboard bins with a weight equal or less than 1 Kg); surgical waste (defined by weight in kg of waste bins and millilitres -ml- of fluids in OR canisters): for this purpose, all bins and canisters allocated in the ORs were monitored and tracked at the end of each procedure, and those in the service area at the end of the daily activities; surgical activity: number of procedures performed each day in each OR; sub-specialty and related procedures: surgical approach (defined as: robotics, laparoscopic/thoracoscopic, endoscopic, conversion or open surgery), operative room time (procedure time: defined by the time in minutes from incision to the end of the procedure, and OR occupation: defined by the time in minutes from patient's admission in the OR to the discharge in the recovery area) estimated blood loss in ml, sub-specialty (colorectal- ileal resections for benign and malignant disease -CRC, liver-hepatobiliary-and pancreatic-HPB, UGI including bariatrics, endocrine, hernias and abdominal wall procedures, thoracic, vascular, urologic, gynaecologic, others), type of surgery (minor - if the procedure time was less or equal 45 minutes - or major - if it was greater than 45 minutes-),^1^ concomitant procedures performed (yes/no), use of XR (yes/no), use of linen drapes (reusable/non-woven fabric/mixed), use of linen gowns (reusable/non-woven fabric/mixed), scrubbing for personnel (water, gel, mixed), number of single use devices disposed in the bins, type of anaesthesia (total intravenous, total gas, balanced, local/spinal), setting (elective, re-allocated/unplanned-emergency), ASA score, classification of the procedure (clean, clean-contaminated and contaminated), and the number of surgeons (including trainees and fellows), anaesthesiologists (including trainees and fellows), and nurses (including scrub and running nurses) for each procedure.

Measurements of the bins (cardboard and polyethylene) were performed with a single electronic scale (LAICA ® PL80281) in the tracked areas as reported in Figure 1 (ORs and service areas), even when they were more (if required by the procedure), or seldomly less. On the other hand, waste bins in patient admission, stock and recovery areas, offices, and other services were not tracked. All other data were collected by the investigators at the end of the procedures and retrieved by the electronic medical records (surgical and anaesthesiologist notes). The number of waste bins tracked during the audit was double-checked with the facility manager of the Health Department for consistency of data. The protocol was submitted and approved by the Health Management Department of the Institution.

***Outcomes of interest.*** The primary outcome of interest was to measure the waste produced in the OR expressed by the weight in kg (for dry products) and the number of cardboard bins at the end of surgical procedures. The weight of surgical waste in each procedure was considered under three different conditions: median weight, net mean weight (given a tare of 0.7 Kg for cardboard bins and 1.9 Kg for polyethylene bins), and weighted mean (considering the number of waste bins/procedure).

The secondary outcomes of interest were: the correlation of the waste with surgical activities and personnel involved, the estimation of the annual waste produced by the most representative sub-specialty, and the estimation of the emissions (CO2 emissions, CO_2_e) and costs. For this purpose, we calculated the emission factors produced by the incineration of medical/clinical waste, as estimated before (1800 kg CO_2_e/ton waste).^2-5^ Costs for waste bin supplies and their incinerations were provided by the Health Management Department and were expressed in euros (1.18 euros kg + 23% VAT= 1.45 euros).

Emissions for bins' transportation were calculated based on Google Maps (from the Institution to the incinerator -270 km-) given 55.9 g CO_2_e/tkm.^6^

***Statistics.*** Categorical data were reported using frequencies and percentages and continuous variables using mean values and standard deviations (SD) or medians and ranges.

Missing data were handled with statistical imputation using a non-parametric Random Forest method whenever possible (missForest package, R software) or mean values (mice package, R software) based on the complexity of the datasets.

Statistical analyses followed a three-step approach: first, correlations between surgical and anaesthesiologic practices/variables and the outcomes of interest were tested using two-tailed univariable and multivariable analyses, and a p-value of 0.05 was considered statistically significant. Quantitative variables were analysed using parametric t-tests and non-parametric Mann–Whitney tests, according to the distribution of variables, whereas qualitative χ2 tests were performed for categorical variables. A generalised linear regression model was used to compute multivariable tests considering dichotomous outcomes (above and below the median value). Odds ratios (OR) and 95% confidence intervals (CI) were calculated for variables providing statistically significant results. Since polyethylene bins were in common areas of adjacent ORs and due to possible re-allocation of procedures, only waste measured in cardboard bins was correlated with the outcomes.

Secondly, we investigated possible causation (relation of cause-effect between variables and the outcomes of interest), as previously performed.^7^ To this end, correlation and partial correlation were identified. Qualitative variables were tested using polychoric and polyserial correlations, whereas Pearson's correlation was used to test quantitative variables (polycor package, R software). Correlations were obtained and defined as strong if ranging between 1.00 and 0.80, medium if between 0.79 and 0.50, moderate if between 0.49 and 0.20, or weak if <0.19. Then, we measured the adequacy and homogeneity of variance sphericity using the Kaiser-Meyer-Olkin and Bartlett's tests in the dataset.

A factor analysis (FA) was then performed to test the complexity and uniqueness of variables (parameters package, R software). These steps allowed the identification of the degrees of collinearity among variables. Finally, a structural equation modelling (SEM) analysis was conducted. All variables were computed in a SEM analysis including multivariable regression, inter-variable correlations, and the measure of direct (of a single variable) and indirect effects (of multiple correlated variables) on the outcomes (Kg of waste and number of waste bins produced) (lavaan package, R software, https://cran.r-project.org/).

Confirmatory factor analysis (CFA) was then conducted, and partial correlations were evaluated with the exclusion of the outcomes.

Thirdly, we calculated costs and emissions for the audit, and based on the estimation of the annual volume of surgeries performed at the Institution using the latest edition of the Italian National Healthcare Outcomes Programme (Piano Nazionale Esiti [PNE]) published in 2023 and reporting data from 2022.^8^

Finally, two models for implementing the management of waste in the OR were tested, the first was based on the idea of reducing cardboard units in each procedure, and the second was developed to test an alternative management of waste bins. This was based on the assumption that the cardboard units are all incinerated (cardboard and waste matter), whereas, after incinerating the bag, a polyethylene bin could be reused 10 times after sterilization, and each cycle of sterilization has an estimated emission of 66–77 g CO2e (average: 71.5 g CO_2_e).^9^ All statistical analyses were performed using R software (https://cran.r-project.org/).

***Disposables and devices.*** The following devices were considered per procedures: single-use devices as mini-invasive/open staplers and related reloads; single-use laparoscopic ports; single-use mini-invasive/open energy devices; plastic covering for scopes and robotic instruments; single-use laparoscopic instruments (i.e. scissors, single-use ligation-clippers, etc).

The following instruments were not considered: robotic arms, drains, needles, and bandages.

**Supplementary Table 1. Details of the waste produced in the OR during the audit**

| DAY OF THE AUDIT (NUMBER OF PROCEDURES) |  | WASTE (Kg) | NET WEIGHT (Kg) | WEIGHTED MEAN (Kg) | Number of BINS | Number of BINS WEIGHT≤1 KG (%) |
| --- | --- | --- | --- | --- | --- | --- |
| DAY 1 (36) |  |  |  |  |  |  |
|  | TOTAL WEIGHT OF THE DAY | 174.8 | 106.9 |  | 97 | 18 (18.5%) |
|  | MEAN WEIGHT OF THE DAY (SD) | 5.5 (2.2) | 3.3 (2.1) | 1.8 (0.6) | 3.0 |  |
|  | MEDIAN WEIGHT OF THE DAY (IQR) | 5.0 (3.5-6.4) | 2.9 (1.8-4.4) | 1.7 (1.3-2.0) | 3.0 |  |
| DAY 2 (35) |  |  |  |  |  |  |
|  | TOTAL WEIGHT OF THE DAY | 202.4 | 133.1 |  | 99 | 7 (7.1%) |
|  | MEAN WEIGHT OF THE DAY (SD) | 6.3 (3.0) | 4.2 (2.7) | 2.0 (0.6) | 3.1 |  |
|  | MEDIAN WEIGHT OF THE DAY (IQR) | 5.6 (4.4-7.0) | 3.7 (2.3-4.9) | 2.0 (1.5-2.4) | 3.0 |  |
| DAY 3 (33) |  |  |  |  |  |  |
|  | TOTAL WEIGHT OF THE DAY | 243.3 | 158.6 |  | 121 | 12 (9.9%) |
|  | MEAN WEIGHT OF THE DAY (SD) | 6.6 | 4.3 | 2.0 | 3.3 |  |
|  | MEDIAN WEIGHT OF THE DAY (IQR) | 5.5 | 3.4 | 1.8 | 3.0 |  |
| DAY 4 (42) |  |  |  |  |  |  |
|  | TOTAL WEIGHT OF THE DAY | 268.9 | 181.4 |  | 125 | 9 (7.2%) |
|  | MEAN WEIGHT OF THE DAY (SD) | 6.6 (3.4) | 4.4 (3.1) | 2.1 (0.8) | 3.0 |  |
|  | MEDIAN WEIGHT OF THE DAY (IQR) | 6.1 (4.6-9.0) | 4.0 (2.5-6.8) | 2.0 (1.5-2.2) | 3.0 |  |
| DAY 5 (33) |  |  |  |  |  |  |
|  | TOTAL WEIGHT OF THE DAY | 254 | 178.4 |  | 108 | 9 (8.3%) |
|  | MEAN WEIGHT OF THE DAY (SD) | 7.7 (2.4) | 5.4 (2.3) | 2.3 (0.7) | 3.3 |  |
|  | MEDIAN WEIGHT OF THE DAY (IQR) | 7 (4.4-7.7) | 4.9 (2.8-5.4) | 2.3 (1.6-2.5) | 3 |  |
| DAY 6 (34) |  |  |  |  |  |  |
|  | TOTAL WEIGHT OF THE DAY | 271.2 | 181.6 |  | 128 | 10 (7.8%) |
|  | MEAN WEIGHT OF THE DAY (SD) | 7.1 (3.8) | 4.8 (3.6) | 2.1 (1.1) | 3.4 |  |
|  | MEDIAN WEIGHT OF THE DAY (IQR) | 6.1 (4.7-7.7) | 3.9 (2.8-5.6) | 2.0 (1.6-2.4) | 3.0 |  |
| DAY 7 (30) |  |  |  |  |  |  |
|  | TOTAL WEIGHT OF THE DAY | 220.8 | 150.1 |  | 101 | 0 (0.0%) |
|  | MEAN WEIGHT OF THE DAY (SD) | 7.4 (2.8) | 5.0 (2.4) | 2.2 (0.6) | 3.4 |  |
|  | MEDIAN WEIGHT OF THE DAY (IQR) | 6.7 (5.3-8.5) | 4.6 (3.2-6.4) | 2.2 (1.7-0.6) | 3.0 |  |
| DAY 8 (41) |  |  |  |  |  |  |
|  | TOTAL WEIGHT OF THE DAY | 257.5 | 175.6 |  | 117 | 5 (4.2%) |
|  | MEAN WEIGHT OF THE DAY (SD) | 7.0 (2.9) | 4.7 (2.3) | 2.2 (0.6) | 3.2 |  |
|  | MEDIAN WEIGHT OF THE DAY (IQR) | 6.7 (5.8-8.2) | 4.6 (3.7-6.1) | 2.1 (1.9-2.5) | 3.0 |  |
| DAY 9 (37) |  |  |  |  |  |  |
|  | TOTAL WEIGHT OF THE DAY | 246.5 | 170.2 |  | 109 | 2 (1.8%) |
|  | MEAN WEIGHT OF THE DAY (SD) | 7.5 (3.7) | 5.2 (3.2) | 2.2 (0.5) | 3.3 |  |
|  | MEDIAN WEIGHT OF THE DAY (IQR) | 6.3 (5.8-8.7) | 4.2 (3.7-6.5) | 2.1 (1.9-2.4) | 3.0 |  |
| DAY 10 (43) |  |  |  |  |  |  |
|  | TOTAL WEIGHT OF THE DAY | 282.7 | 193.1 |  | 128 | 11 (8.6%) |
|  | MEAN WEIGHT OF THE DAY (SD) | 7.1 (3.0) | 4.8 (2.7) | 2.2 (0.7) | 3.2 |  |
|  | MEDIAN WEIGHT OF THE DAY (IQR) | 6.5 (4.6-8.0) | 4.4 (2.5-5.9) | 2.1 (1.6-2.5) | 3.0 |  |
| OVERALL AUDIT |  |  |  |  |  |  |
| DAY 1-10 (364) |  | 2422.1 | 1629 |  | 1133 | 83 (7.3%) |

**Supplementary Table 2. Data collected during the audit**

| Variable s | | | | |
| --- | --- | --- | --- | --- |
| Drapes | **N (%)** | | | |
|  | REUSABLE 291 (79.9%) | | | |
|  | MIXED (REUSABLE AND NON-WOVEN FABRIC) 41 (12.3%) | | | |
|  | NON-WOVEN FABRIC 17 (4.7%) | | | |
|  | MISSING 15 (4.2%) | | | |
| Gowns | **N (%)** | | | |
|  | REUSABLE 278 (76.4%) | | | |
|  | MIXED (REUSABLE AND NON-WOVEN FABRIC) 66 (18.1%) | | | |
|  | NON-WOVEN FABRIC 5 (1.4%) | | | |
|  | MISSING 15 (4.2%) | | | |
| Scrubbing for Personnel | **N (%)** | | | |
|  | GEL 106 (29.1%) | | | |
|  | MIXED (GEL AND WATER) 233 (64.0%) | | | |
|  | WATER 3 (0.8%) | | | |
|  | MISSING 22 (6.1%) | | | |
| Setting | **N (%)** | | | |
|  | ELECTIVE 360 (98.9%) | | | |
|  | EMERGENCY 4 (1.1%) | | | |
|  | MISSING 0 (0.0%) | | | |
| Sub-specialty | **N (%)** | | | |
|  | CRC/ILEAL 57 (15.6%) | | | |
|  | ENDOCRINE 46 (12.6%) | | | |
|  | GYNECOLOGY 46 (12.6%) | | | |
|  | UGI/BARIATRICS 44 (12.1%) | | | |
|  | ABDOMINAL WALL/HERNIA 21 (5.7%) | | | |
|  | HPB 35 (9.6%) | | | |
|  | THORACIC 34 (9.3%) | | | |
|  | UROLOGY 42 (11.6%) | | | |
|  | VASCULAR 13 (3.6%) | | | |
|  | OTHER 26 (7.1%) | | | |
|  | MISSING 0 (0.0%) | | | |
| Surgical Approach | **N (%)** | | | |
|  | LAPAROSCOPY/THORACOSCOPY 149 (40.9%) | | | |
|  | OPEN 141 (38.7%) | | | |
|  | ROBOTICS 42 (11.5%) | | | |
|  | ENDOSCOPIC 18 (4.9%) | | | |
|  | CONVERSION 14 (3.8%) | | | |
|  | MISSING 0 (0.0%) | | | |
| Conversion | **N (%)** | | | |
|  | NO 348 (95.6%) | | | |
|  | YES 16 (4.4%) | | | |
|  | MISSING (0.0%) | | | |
| SINGLE USE DISPOSABLE DEVICES (NUMBER) /pROCEDURE | **MISSING RATE (%)** | **MEAN (SD)** | **MEDIAN (IQR)** | **HISTOGRAM** |
|  | 0% | 4.9 (4.5) | 4 (1-7) | ▇▂▁▁▁ |
| Classification of THE Procedure | **N (%)** |  |  |  |
|  | CLEAN 221 (60.7%) | | | |
|  | CLEAN-CONTAMINATED 127 (34.8%) | | | |
|  | CONTAMINATED 8 (2.2%) | | | |
|  | MISSING 8 (2.2%) | | | |
| ASA Score | **N (%)** | | | |
|  | ASA 1 28 (7.7%) | | | |
|  | ASA 2 207 (56.8%) | | | |
|  | ASA 3 118 (32.5%) | | | |
|  | ASA 4 1 (0.27%) | | | |
|  | MISSING 10 (2.7%) | | | |
| Type of Anesthesia | **N (%)** | | | |
|  | BALANCED 320 (87.9%) | | | |
|  | TOTAL GAS 9 (2.5%) | | | |
|  | LOCAL 7 (1.9%) | | | |
|  | TOTAL IV 5 (1.4%) | | | |
|  | SPINAL 4 (1.1%) | | | |
|  | MISSING 19 (5.2%) | | | |
| Procedures | **N (%)** | | | |
|  | MAJOR 288 (79.1%) | | | |
|  | MINOR 67 (18.4%) | | | |
|  | MISSING 9 (2.5%) | | | |
| Procedure Re-allocated/Unplanned | **N (%)** | | | |
|  | NO 326 (89.6%) | | | |
|  | YES 38 (10.4%) | | | |
|  | MISSING 0 (0.0%) | | | |
| XR use | **N (%)** | | | |
|  | NO 348 (95.6%) | | | |
|  | YES 15 (4.1%) | | | |
|  | MISSING 1 (0.3%) | | | |
| Multi-organ or concomitant procedures | **N (%)** | | | |
|  | NO 334 (91.7%) | | | |
|  | YES 27 (7.4%) | | | |
|  | MISSING 3 (0.9%) | | | |
| PROCEDURE TIME (MINUTES)/procedure | **MISSING RATE (%)** | **MEAN (SD)** | **MEDIAN (IQR)** | **HISTOGRAM** |
|  | 2.5% | 137.6 (122.8) | 100 (54-186.5) | ▇▂▁▁▁ |
| TIME OR OCCUPATION (MINUTES)/procedure | **MISSING RATE (%)** | **MEAN (SD)** | **MEDIAN (IQR)** | **HISTOGRAM** |
|  | 2.5% | 189.0 (137.1) | 153 (90.5-248) | ▇▃▁▁▁ |
| DISCARDED FLUIDS (ml)/procedure | **MISSING RATE (%)** | **MEAN (SD)** | **MEDIAN (IQR)** | **HISTOGRAM** |
|  | 0.6% | 781.9 (1719.5) | 125 (0-800) | ▇▁▁▁▁ |
| BLOOD LOSS (ML)/PROCEDURE | **MISSING RATE (%)** | **MEAN (SD)** | **MEDIAN (IQR)** | **HISTOGRAM** |
|  | 1.4% | 70.1 (223.3) | 0 (0-50) | ▇▁▁▁▁ |
| SURGEONS (NUMBER)/PROCEDURE | **MISSING RATE (%)** | **MEAN (SD)** | **MEDIAN (IQR)** | **HISTOGRAM** |
|  | 0% | 3.1 (0.86) | 3 (3-4) | ▃▇▃▁▁ |
| ANESTHESIOLOGISTS (NUMBER)/PROCEDURE | **MISSING RATE (%)** | **MEAN (SD)** | **MEDIAN (IQR)** | **HISTOGRAM** |
|  | 0% | 1.7 (0.54) | 2 (1-2) | ▃▇▁▁▁ |
| NURSES (NUMBER)/PROCEDURE | **MISSING RATE (%)** | **MEAN (SD)** | **MEDIAN (IQR)** | **HISTOGRAM** |
|  | 0% | 2.3 (0.60) | 2 (1-2) | ▁▇▂▁▁ |
| WASTE (Kg)/PROCEDURE | **MISSING RATE (%)** | **MEAN (SD)** | **MEDIAN (IQR)** | **HISTOGRAM** |
|  | 5.3% | 6.8 (3.1) | 6.2 (4.6-8.2) | ▇▅▂▁▁ |
| NET WEIGHT (kg)/PROCEDURE | **MISSING RATE (%)** | **MEAN (SD)** | **MEDIAN (IQR)** | **HISTOGRAM** |
|  | 5.3% | 4.6 (2.7) | 4.1 (2.6-5.9) | ▇▅▁▁▁ |
| WEIGHTED MEAN BINS (Kg)/PROCEDURE | **MISSING RATE (%)** | **MEAN (SD)** | **MEDIAN (IQR)** | **HISTOGRAM** |
|  | 5.3% | 2.1 (0.71) | 2.0 (1.6-2.5) | ▇▅▁▁▁ |
| BINS (NUMBER)/PROCEDURE | **MISSING RATE (%)** | **MEAN (SD)** | **MEDIAN (IQR)** | **HISTOGRAM** |
|  | 5.3% | 3.2 (0.7) | 3 (3-3) | ▁▇▁▁▁ |
| BINS WEIGHT≤1 KG (NUMBER)/PROCEDURE | **MISSING RATE (%)** | **MEAN (SD)** | **MEDIAN (IQR)** | **HISTOGRAM** |
|  | 5.3% | 0.2 (0.51) | 0 (0-0) | ▇▁▂▁▁ |

**^HPB:^** ^Hepatobiliary-Pancreatic;^ **^UGI:^** ^Upper Gastrointestinal^

**Supplementary Table 3. Polyethylene bins collected during the audit**

|  | WASTE (Kg) | NET WEIGHT (Kg) | NUMBER OF BINS (%) |
| --- | --- | --- | --- |
| DAY 1  (36 PROCEDURES) |  |  |  |
| BINS WEIGHT (TOTAL) | 58.5 | 45.2 | TRACKED: 8 (66.7%) |
|  |  |  | MISSING: 4 (33.3%) |
| DAY 2  (35 PROCEDURES) |  |  |  |
| BINS WEIGHT (TOTAL) | 0.0 | 0.0 | TRACKED: 0 (0.0%) |
|  |  |  | MISSING: 12 (100.0%) |
| DAY 3  (33 PROCEDURES) |  |  |  |
| BINS WEIGHT (TOTAL) | 65.5 | 50.3 | TRACKED: 8 (66.7%) |
|  |  |  | MISSING: 4 (33.3%) |
| DAY 4  (42 PROCEDURES) |  |  |  |
| BINS WEIGHT (TOTAL) | 121.3 | 98.5 | TRACKED: 12 (100.0%) |
|  |  |  | MISSING: 0 (0.0%) |
| DAY 5  (33 PROCEDURES) |  |  |  |
| BINS WEIGHT (TOTAL) | 91.4 | 82.4 | TRACKED: 10 (83.3%) |
|  |  |  | MISSING: 2 (16.7%) |
| DAY 6  (34 PROCEDURES) |  |  |  |
| BINS WEIGHT (TOTAL) | 90.1 | 81.1 | TRACKED: 10 (83.3%) |
|  |  |  | MISSING: 2 (16.7%) |
| DAY 7  (30 PROCEDURES) |  |  |  |
| BINS WEIGHT (TOTAL) | 67.2 | 61.8 | TRACKED: 6 (50.0%) |
|  |  |  | MISSING: 6 (50.0%) |
| DAY 8  (41 PROCEDURES) |  |  |  |
| BINS WEIGHT (TOTAL) | 104.8 | 96.7 | TRACKED: 9 (75.0%) |
|  |  |  | MISSING: 3 (25.0%) |
| DAY 9  (37 PROCEDURES) |  |  |  |
| BINS WEIGHT (TOTAL) | 97.4 | 87.5 | TRACKED: 11 (91.7%) |
|  |  |  | MISSING: 1 (8.3%) |
| DAY 10  (43 PROCEDURES) |  |  |  |
| BINS WEIGHT (TOTAL) | 127.7 | 117.8 | TRACKED: 11 (91.7%) |
|  |  |  | MISSING: 1 (8.3%) |
| OVERALL AUDIT: day 1-10  (364 procedures) |  |  |  |
| BINS WEIGHT (TOTAL) | 823.9 | 721.3 | TRACKED: 85 (70.8%) |
|  |  |  | MISSING: 35 (29.2%) |

**Supplementary Table 4. Re-allocated/unplanned procedures**

| SUB-SPECIALTY | N | % |
| --- | --- | --- |
| ABDOMINAL WALL/HERNIA | 2 | 5.3 |
| COLORECTAL/ILEAL | 4 | 10.5 |
| ENDOCRINE | 6 | 15.8 |
| GYNAECOLOGY | 3 | 7.9 |
| HPB | 3 | 7.9 |
| OTHER | 5 | 13.2 |
| THORACIC | 1 | 2.6 |
| UGI/BARIATRICS | 4 | 10.5 |
| UROLOGY | 8 | 21.1 |
| VASCULAR | 2 | 5.3 |
| TOTAL | 38 | 100 |

**^HPB:^** ^Hepatobiliary-Pancreatic;^ **^UGI:^** ^Upper Gastrointestinal^

**Supplementary Table 5. Univariable analysis for the outcomes of interest: median waste/procedure and median number of bins/procedure**

| Covariate | VARIABLE REFERENCE | MEDIAN WEIGHT - KG (IQR) | p-value | MEDIAN NUMBER OF BINS (IQR) | p-value |
| --- | --- | --- | --- | --- | --- |
| DRAPES | | | | | |
|  | REUSABLE | 6.2 (4.6 to 7.6) | **<0.001** | 3.0 (3.0 to 3.0) | 0.933 |
|  | MIXED | 8.1 (5.9 to 10.2) |  | 3.0 (3.0 to 3.5) |  |
|  | NON-WOVEN FABRIC | 8.7 (6.2 to 11.6) |  | 3.0 (3.0 to 3.4) |  |
| GOWNS | | | | | |
|  | REUSABLE | 6.2 (4.6 to 7.5) | **<0.001** | 3.0 (3.0 to 3.0) | **0.001** |
|  | MIXED | 7.8 (5.5 to 10.9) |  | 3.0 (3.0 to 4.0) |  |
|  | NON-WOVEN FABRIC | 11.6 (9.9 to 11.8) |  | 3.0 (3.0 to 3.0) |  |
| SCRUBBING FOR PERSONNEL | | | | | |
|  | GEL | 6.1 (4.8 to 8.3) | 0.728 | 3.0 (3.0 to 3.0) | 0.335 |
|  | MIXED | 6.5 (4.6 to 8.2) |  | 3.0 (3.0 to 3.0) |  |
|  | WATER | 6.6 (6.1 to 10.3) |  | 3.0 (3.0 to 4.0) |  |
| SETTING | | | | | |
|  | ELECTIVE | 6.3 (4.7 to 8.2) | 0.421 | 3.0 (3.0 to 3.0) | 0.868 |
|  | EMERGENCY | 6.9 (6.3 to 7.9) |  | 3.0 (3.0 to 3.1) |  |
| SUB-SPECIALTY | | | | | |
|  | ABDOMINAL WALL/HERNIA | 4.5 (3.9 to 5.4) | **<0.001** | 3.0 (3.0 to 3.0) | **<0.001** |
|  | COLORECTAL/ILEAL | 7.3 (6.3 to 9.4) |  | 3.0 (3.0 to 4.0) |  |
|  | ENDOCRINE | 4.4 (3.5 to 6.2) |  | 3.0 (3.0 to 3.0) |  |
|  | GYNAECOLOGY | 6.5 (5.9 to 8.7) |  | 3.0 (3.0 to 3.0) |  |
|  | HPB | 8.4 (5.0 to 12.6) |  | 3.5 (3.0 to 4.0) |  |
|  | OTHER | 6.3 (4.2 to 7.4) |  | 3.0 (3.0 to 3.0) |  |
|  | THORACIC | 6.5 (5.7 to 7.4) |  | 3.0 (3.0 to 3.0) |  |
|  | UGI/BARIATRICS | 5.8 (4.9 to 7.2) |  | 3.0 (3.0 to 3.0) |  |
|  | UROLOGY | 6.6 (4.6 to 8.2) |  | 3.0 (3.0 to 3.1) |  |
|  | VASCULAR | 6.1 (5.0 to 10.6) |  | 3.0 (3.0 to 4.0) |  |
| SURGICAL APPROACH | | | | | |
|  | CONVERSION | 8.9 (7.2 to 10.5) | **<0.001** | 4.0 (3.0 to 4.0) | **0.005** |
|  | ENDOSCOPIC | 5.7 (4.3 to 7.5) |  | 3.0 (3.0 to 3.0) |  |
|  | LAPAROSCOPY/THORACOSCOPY | 6.3 (5.2 to 7.6) |  | 3.0 (3.0 to 3.0) |  |
|  | OPEN | 5.4 (4.2 to 7.2) |  | 3.0 (3.0 to 3.0) |  |
|  | ROBOTICS | 8.5 (7.3 to 11.8) |  | 3.0 (3.0 to 3.8) |  |
| CONVERSION | | | | | |
|  | NO | 6.2 (4.7 to 8.1) | **0.001** | 3.0 (3.0 to 3.0) | **0.002** |
|  | YES | 8.9 (7.1 to 11.1) |  | 4.0 (3.0 to 4.0) |  |
| SINGLE USE DISPOSABLE DEVICES (NUMBER) | | | | | |
|  | [0.0.25.0] | 6.3 (4.8 to 8.2) | **<0.001** | 3.0 (3.0 to 3.0) | **<0.001** |
| CLASSIFICATION OF THE PROCEDURE | | | | | |
|  | CLEAN | 5.8 (4.3 to 7.3) | **<0.001** | 3.0 (3.0 to 3.0) | 0.056 |
|  | CLEAN-CONTAMINATED | 7.1 (6.1 to 9.3) |  | 3.0 (3.0 to 3.0) |  |
|  | CONTAMINATED | 7.7 (5.8 to 10.6) |  | 3.0 (3.0 to 4.0) |  |
| ASA SCORE | | | | | |
|  | ASA 1 | 5.7 (4.5 to 7.0) | 0.421 | 3.0 (3.0 to 3.0) | 0.941 |
|  | ASA 2 | 6.3 (4.8 to 8.0) |  | 3.0 (3.0 to 3.0) |  |
|  | ASA 3 | 6.6 (4.8 to 9.4) |  | 3.0 (3.0 to 3.0) |  |
| TYPE OF ANESTHESIA | | | | | |
|  | BALANCED | 6.4 (4.8 to 8.2) | 0.239 | 3.0 (3.0 to 3.0) | 0.87 |
|  | LOCAL | 4.2 (3.6 to 7.5) |  | 3.0 (3.0 to 3.0) |  |
|  | SPINAL | 5.3 (4.3 to 6.4) |  | 3.0 (3.0 to 3.0) |  |
|  | TOTAL GAS | 6.2 (5.7 to 9.6) |  | 3.0 (3.0 to 3.0) |  |
|  | TOTAL IV | 4.6 (4.5 to 4.8) |  | 3.0 (3.0 to 3.0) |  |
| PROCEDURE | | | | | |
|  | MAJOR | 6.6 (5.2 to 8.5) | **<0.001** | 3.0 (3.0 to 3.0) | **0.04** |
|  | MINOR | 4.6 (3.6 to 6.6) |  | 3.0 (3.0 to 3.0) |  |
| Procedure Re-allocated/Unplanned | | | | | |
|  | NO | 6.4 (4.9 to 8.4) | **0.014** | 3.0 (3.0 to 3.0) | **0.294** |
|  | YES | 5.4 (4.1 to 6.8) |  | 3.0 (3.0 to 3.0) |  |
| XR USE | | | | | |
|  | NO | 6.3 (4.8 to 8.2) | 0.57 | 3.0 (3.0 to 3.0) | 0.391 |
|  | YES | 7.6 (4.6 to 8.9) |  | 3.0 (3.0 to 3.5) |  |
| Multi-organ or concomitant procedures | | | | | |
|  | NO | 6.2 (4.6 to 8.0) | **<0.001** | 3.0 (3.0 to 3.0) | **<0.001** |
|  | YES | 9.5 (7.2 to 10.9) |  | 4.0 (3.0 to 4.0) |  |
| PROCEDURE TIME (MINUTES) | | | | | |
|  | [15.0.755.0] | 6.3 (4.8 to 8.2) | **<0.001** | 3.0 (3.0 to 3.0) | **<0.001** |
| TIME OR OCCUPATION (MINUTES) | | | | | |
|  | [38.0.824.0] | 6.3 (4.8 to 8.2) | **<0.001** | 3.0 (3.0 to 3.0) | **<0.001** |
| DISCARDED FLUIDS (ML) | | | | | |
|  | [0.0.17000.0] | 6.3 (4.8 to 8.2) | **<0.001** | 3.0 (3.0 to 3.0) | **0.004** |
| BLOOD LOSS (ML) | | | | | |
|  | [0.0.2500.0] | 6.3 (4.8 to 8.2) | **<0.001** | 3.0 (3.0 to 3.0) | **<0.001** |
| SURGEONS (NUMBER) | | | | | |
|  | [1.0.7.0] | 6.3 (4.8 to 8.2) | **<0.001** | 3.0 (3.0 to 3.0) | **<0.001** |
| ANESTHESIOLOGISTS (NUMBER) | | | | | |
|  | [1.0.5.0] | 6.3 (4.8 to 8.2) | 0.109 | 3.0 (3.0 to 3.0) | 0.451 |
| NURSES (NUMBER) | | | | | |
|  | [1.0.5.0] | 6.3 (4.8 to 8.2) | **<0.001** | 3.0 (3.0 to 3.0) | **<0.001** |

**^HPB:^** ^Hepatobiliary-Pancreatic;^ **^UGI:^** ^Upper Gastrointestinal^

**Supplementary Table 6. Univariable analysis for the outcome of interest median net waste/procedure**

| COVARIATE | VARIABLE REFERENCE | MEDIAN WEIGHT (IQR) | p-value |
| --- | --- | --- | --- |
| DRAPES | | | |
|  | **REUSABLE** | 4.0 (2.6 to 5.4) | **<0.001** |
|  | **MIXED** | 5.7 (3.8 to 7.4) |  |
|  | **NON-WOVEN FABRIC** | 6.4 (4.1 to 8.9) |  |
| GOWNS | | | |
|  | **REUSABLE** | 4.0 (2.5 to 5.3) | **<0.001** |
|  | **MIXED** | 5.5 (3.4 to 8.6) |  |
|  | **NON-WOVEN FABRIC** | 9.0 (7.8 to 9.5) |  |
| SCRUBBING FOR PERSONNEL | | | |
|  | **GEL** | 4.0 (2.8 to 5.9) | 0.717 |
|  | **MIXED** | 4.2 (2.6 to 5.9) |  |
|  | **WATER** | 4.5 (4.0 to 7.5) |  |
| SETTING | | | |
|  | **ELECTIVE** | 4.2 (2.7 to 5.9) | 0.334 |
|  | **EMERGENCY** | 4.9 (4.2 to 6.0) |  |
| SUB-SPECIALTY | | | |
|  | **ABDOMINAL WALL/HERNIA** | 2.4 (1.8 to 3.3) | **<0.001** |
|  | **COLORECTAL/ILEAL** | 4.9 (4.2 to 6.6) |  |
|  | **ENDOCRINE** | 2.4 (1.5 to 4.1) |  |
|  | **GYNAECOLOGY** | 4.6 (3.8 to 6.6) |  |
|  | **HPB** | 5.4 (3.0 to 9.8) |  |
|  | **OTHER** | 4.2 (2.2 to 5.3) |  |
|  | **THORACIC** | 4.2 (3.6 to 5.2) |  |
|  | **UGI/BARIATRICS** | 3.7 (3.1 to 5.1) |  |
|  | **UROLOGY** | 4.5 (2.5 to 5.9) |  |
|  | **VASCULAR** | 4.0 (2.9 to 8.5) |  |
| SURGICAL APPROACH | | | |
|  | **CONVERSION** | 6.5 (4.8 to 7.7) | **<0.001** |
|  | **ENDOSCOPIC** | 3.6 (2.4 to 5.4) |  |
|  | **LAPAROSCOPY/THORACOSCOPY** | 4.2 (3.2 to 5.4) |  |
|  | **OPEN** | 3.3 (2.1 to 5.1) |  |
|  | **ROBOTICS** | 6.4 (5.2 to 9.4) |  |
| CONVERSION | | | |
|  | **NO** | 4.1 (2.6 to 5.7) | **0.001** |
|  | **YES** | 6.5 (4.6 to 7.9) |  |
| SINGLE USE DISPOSABLE DEVICES (NUMBER) | | | |
|  | **[0.0.25.0]** | 4.2 (2.7 to 5.9) | **<0.001** |
| CLASSIFICATION OF THE PROCEDURE | | | |
|  | **CLEAN** | 3.6 (2.3 to 5.2) | **<0.001** |
|  | **CLEAN-CONTAMINATED** | 4.8 (4.0 to 6.8) |  |
|  | **CONTAMINATED** | 5.2 (3.6 to 7.9) |  |
| ASA SCORE | | | |
|  | **ASA 1** | 3.6 (2.4 to 4.6) | 0.294 |
|  | **ASA 2** | 4.2 (2.8 to 5.6) |  |
|  | **ASA 3** | 4.3 (2.7 to 6.9) |  |
| TYPE OF ANESTHESIA | | | |
|  | **BALANCED** | 4.2 (2.9 to 5.9) | 0.222 |
|  | **LOCAL** | 2.1 (1.7 to 5.0) |  |
|  | **SPINAL** | 3.2 (2.2 to 4.3) |  |
|  | **TOTAL GAS** | 4.1 (3.6 to 7.4) |  |
|  | **TOTAL IV** | 2.5 (2.4 to 2.7) |  |
| PROCEDURE | | | |
|  | **MAJOR** | 4.4 (3.2 to 6.3) | **<0.001** |
|  | **MINOR** | 2.5 (1.7 to 4.4) |  |
| PROCEDURE RE-ALLOCATED/UNPLANNED | | | |
|  | **NO** | 4.2 (2.9 to 6.0) | **0.011** |
|  | **YES** | 3.3 (2.0 to 4.7) |  |
| XR USE | | | |
|  | **NO** | 4.2 (2.7 to 5.8) | 0.583 |
|  | **YES** | 5.5 (2.5 to 6.6) |  |
| MULTI-ORGANS OR CONCOMITANT PROCEDURES | | | |
|  | **NO** | 4.1 (2.5 to 5.5) | **<0.001** |
|  | **YES** | 7.3 (5.2 to 8.4) |  |
| PROCEDURE TIME (MINUTES) | | | |
|  | **[15.0.755.0]** | 4.2 (2.7 to 5.9) | **<0.001** |
| TIME OR OCCUPATION (MINUTES) | | | |
|  | **[38.0.824.0]** | 4.2 (2.7 to 5.9) | **<0.001** |
| DISCARDED FLUIDS (ML) | | | |
|  | **[0.0.17000.0]** | 4.2 (2.7 to 5.9) | **<0.001** |
| BLOOD LOSS (ML) | | | |
|  | **[-0.0.2500.0]** | 4.2 (2.7 to 5.9) | **<0.001** |
| SURGEONS (NUMBER) | | | |
|  | **[1.0.7.0]** | 4.2 (2.7 to 5.9) | **<0.001** |
| ANESTHESIOLOGISTS (NUMBER) | | | |
|  | **[1.0.5.0]** | 4.2 (2.7 to 5.9) | 0.082 |
| NURSES (NUMBER) | | | |
|  | **[1.0.5.0]** | 4.2 (2.7 to 5.9) | **<0.001** |

**^HPB:^** ^Hepatobiliary-Pancreatic;^ **^UGI:^** ^Upper Gastrointestinal^

**Supplementary Table 7. Univariable analysis for the outcome of interest median weighted mean waste/procedure**

| COVARIATE | VARIABLE REFERENCE | MEDIAN WEIGHT (IQR) | p-value |
| --- | --- | --- | --- |
| DRAPES | | | |
|  | **REUSABLE** | 2.0 (1.6 to 2.4) | **<0.001** |
|  | **MIXED** | 2.5 (1.9 to 3.1) |  |
|  | **NON-WOVEN FABRIC** | 2.6 (2.0 to 3.2) |  |
| GOWNS | | | |
|  | **REUSABLE** | 2.0 (1.6 to 2.4) | **<0.001** |
|  | **MIXED** | 2.3 (1.9 to 2.8) |  |
|  | **NON-WOVEN FABRIC** | 3.3 (3.0 to 3.9) |  |
| SCRUBBING FOR PERSONNEL | | | |
|  | **GEL** | 2.0 (1.7 to 2.5) | 0.814 |
|  | **MIXED** | 2.1 (1.7 to 2.5) |  |
|  | **WATER** | 2.2 (2.0 to 2.5) |  |
| SETTING | | | |
|  | **ELECTIVE** | 2.0 (1.7 to 2.5) | 0.17 |
|  | **EMERGENCY** | 2.4 (2.1 to 2.9) |  |
| SUB-SPECIALTY | | | |
|  | **ABDOMINAL WALL/HERNIA** | 1.5 (1.3 to 1.7) | **<0.001** |
|  | **COLORECTAL/ILEAL** | 2.3 (1.9 to 2.7) |  |
|  | **ENDOCRINE** | 1.5 (1.2 to 2.0) |  |
|  | **GYNAECOLOGY** | 2.2 (2.0 to 2.8) |  |
|  | **HPB** | 2.3 (1.7 to 2.8) |  |
|  | **OTHER** | 1.9 (1.5 to 2.2) |  |
|  | **THORACIC** | 2.1 (1.8 to 2.4) |  |
|  | **UGI/BARIATRICS** | 2.0 (1.7 to 2.3) |  |
|  | **UROLOGY** | 2.2 (1.7 to 2.6) |  |
|  | **VASCULAR** | 2.0 (1.7 to 2.2) |  |
| SURGICAL APPROACH | | | |
|  | **CONVERSION** | 2.5 (2.0 to 2.8) | **<0.001** |
|  | **ENDOSCOPIC** | 1.9 (1.6 to 2.3) |  |
|  | **LAPAROSCOPY/THORACOSCOPY** | 2.1 (1.8 to 2.4) |  |
|  | **OPEN** | 1.8 (1.5 to 2.2) |  |
|  | **ROBOTICS** | 2.7 (2.3 to 3.2) |  |
| CONVERSION | | | |
|  | **NO** | 2.0 (1.6 to 2.5) | **0.011** |
|  | **YES** | 2.5 (2.1 to 2.8) |  |
| SINGLE USE DISPOSABLE DEVICES (NUMBER) | | | |
|  | **[0.0.25.0]** | 2.0 (1.7 to 2.5) | **<0.001** |
| CLASSIFICATION OF THE PROCEDURE | | | |
|  | **CLEAN** | 1.9 (1.5 to 2.3) | **<0.001** |
|  | **CLEAN-CONTAMINATED** | 2.3 (2.0 to 2.7) |  |
|  | **CONTAMINATED** | 2.2 (1.9 to 2.5) |  |
| ASA SCORE | | | |
|  | **ASA 1** | 1.8 (1.5 to 2.2) | 0.154 |
|  | **ASA 2** | 2.0 (1.6 to 2.5) |  |
|  | **ASA 3** | 2.1 (1.7 to 2.6) |  |
| TYPE OF ANESTHESIA | | | |
|  | **BALANCED** | 2.0 (1.7 to 2.5) | 0.165 |
|  | **LOCAL** | 1.6 (1.3 to 2.2) |  |
|  | **SPINAL** | 1.8 (1.4 to 2.1) |  |
|  | **TOTAL GAS** | 2.1 (1.9 to 2.5) |  |
|  | **TOTAL IV** | 1.5 (1.5 to 1.6) |  |
| PROCEDURE | | | |
|  | **MAJOR** | 2.1 (1.7 to 2.6) | **<0.001** |
|  | **MINOR** | 1.7 (1.3 to 2.1) |  |
| PROCEDURE RE-ALLOCATED/UNPLANNED | | | |
|  | **NO** | 2.1 (1.7 to 2.5) | **0.014** |
|  | **YES** | 1.7 (1.4 to 2.2) |  |
| XR USE | | | |
|  | **NO** | 2.0 (1.7 to 2.5) | 0.768 |
|  | **YES** | 2.1 (1.7 to 2.6) |  |
| MULTI-ORGANS OR CONCOMITANT PROCEDURES | | | |
|  | **NO** | 2.0 (1.6 to 2.4) | **<0.001** |
|  | **YES** | 2.6 (2.4 to 2.9) |  |
| PROCEDURE TIME (MINUTES) | | | |
|  | **[15.0.755.0]** | 2.0 (1.7 to 2.5) | **<0.001** |
| TIME OR OCCUPATION (MINUTES) | | | |
|  | **[38.0.824.0]** | 2.0 (1.7 to 2.5) | **<0.001** |
| DISCARDED FLUIDS (ML) | | | |
|  | **[0.0.17000.0]** | 2.0 (1.7 to 2.5) | **<0.001** |
| BLOOD LOSS (ML) | | | |
|  | **[-0.0.2500.0]** | 2.0 (1.7 to 2.5) | **<0.001** |
| SURGEONS (NUMBER) | | | |
|  | **[1.0.7.0]** | 2.0 (1.7 to 2.5) | **<0.001** |
| ANESTHESIOLOGISTS (NUMBER) | | | |
|  | **[1.0.5.0]** | 2.0 (1.7 to 2.5) | 0.037 |
| NURSES (NUMBER) | | | |
|  | **[1.0.5.0]** | 2.0 (1.7 to 2.5) | **<0.001** |

**^HPB:^** ^Hepatobiliary-Pancreatic;^ **^UGI:^** ^Upper Gastrointestinal^

**Supplementary Table 8. Generalised Linear Regression Models**

| **OUTCOME: WASTE** |  |  |  |  |
| --- | --- | --- | --- | --- |
| **COEFFICIENTS** | **ESTIMATE** | **STD. ERROR** | **Z-VALUE** | **PR(>\|Z\|)** |
| (Intercept) | -1.57E+01 | 8.71E+02 | -0.018 | 0.98565 |
| **DRAPES** |  |  |  |  |
| MIXED | 6.38E-01 | 5.59E-01 | 1.142 | 0.25341 |
| NON-WOVEN FABRIC | 1.07E+00 | 9.05E-01 | 1.177 | 0.23926 |
| **GOWNS** |  |  |  |  |
| MIXED | -2.98E-01 | 4.50E-01 | -0.663 | 0.5072 |
| NON-WOVEN FABRIC | -9.30E-01 | 1.65E+00 | -0.564 | 0.57263 |
| **SUB-SPECIALTY** |  |  |  |  |
| COLORECTAL/ILEAL | 1.19E+00 | 8.03E-01 | 1.484 | 0.13778 |
| ENDOCRINE | 3.95E-01 | 7.82E-01 | 0.506 | 0.61315 |
| GYNAECOLOGY | 3.38E-02 | 8.30E-01 | 0.041 | 0.96756 |
| HPB | 1.20E+00 | 8.58E-01 | 1.395 | 0.16294 |
| OTHER | 1.81E+00 | 8.04E-01 | 2.25 | **0.02444** |
| THORACIC | 1.67E+00 | 7.85E-01 | 2.127 | **0.03342** |
| UGI/BARIATRICS | 5.32E-01 | 8.29E-01 | 0.642 | 0.52076 |
| UROLOGY | 1.53E+00 | 8.77E-01 | 1.741 | 0.08161 |
| VASCULAR | 2.97E-01 | 9.56E-01 | 0.311 | 0.75612 |
| **SURGICAL APPROACH** |  |  |  |  |
| ENDOSCOPIC | 9.84E+00 | 8.71E+02 | 0.011 | 0.99099 |
| LAPAROSCOPY/THORACOSCOPY | 1.10E+01 | 8.71E+02 | 0.013 | 0.98991 |
| OPEN | 1.07E+01 | 8.71E+02 | 0.012 | 0.99022 |
| ROBOTICS | 1.30E+01 | 8.71E+02 | 0.015 | 0.98812 |
| **CONVERSION (YES)** | 1.26E+01 | 8.71E+02 | 0.014 | 0.98846 |
| **SINGLE USE DISPOSABLE DEVICES (NUMBER)** | -1.33E-02 | 4.41E-02 | -0.302 | 0.76257 |
| **CLASSIFICATION OF THE PROCEDURE** |  |  |  |  |
| CLEAN-CONTAMINATED | 1.14E+00 | 4.15E-01 | 2.755 | **0.00588** |
| CONTAMINATED | -2.32E-01 | 9.36E-01 | -0.248 | 0.80429 |
| **PROCEDURE (MINOR)** | 5.21E-01 | 4.17E-01 | 1.249 | 0.21173 |
| **PROCEDURE RE-ALLOCATED/UNPLANNED (YES)** | 3.02E-01 | 4.33E-01 | 0.696 | 0.48625 |
| **MULTI-ORGAN OR CONCOMITANT PROCEDURES (YES)** | 8.10E-01 | 6.96E-01 | 1.164 | 0.2443 |
| **PROCEDURE TIME (MINUTES)** | 9.69E-03 | 5.10E-03 | 1.9 | 0.05749 |
| **TIME OR OCCUPATION (MINUTES)** | -1.77E-03 | 4.10E-03 | -0.432 | 0.66589 |
| **DISCARDED FLUIDS (ML)** | -4.29E-06 | 9.40E-05 | -0.046 | 0.96364 |
| **BLOOD LOSS (ML)** | 1.33E-03 | 1.01E-03 | 1.314 | 0.18881 |
| **SURGEONS (NUMBER)** | 3.13E-01 | 2.26E-01 | 1.382 | 0.16709 |
| **NURSES (NUMBER)** | 5.52E-01 | 2.66E-01 | 2.074 | **0.03809** |
| **OUTCOME: NUMBER OF BINS** |  |  |  |  |
| **COEFFICIENTS** | **ESTIMATE** | **STD. ERROR** | **Z-VALUE** | **PR(>\|Z\|)** |
| (Intercept) | -6.03013 | 2.279784 | -2.645 | 0.00817 |
| **GOWNS** |  |  |  |  |
| MIXED | 0.523042 | 0.423786 | 1.234 | 0.21712 |
| NON-WOVEN FABRIC | 0.548804 | 1.356028 | 0.405 | 0.68569 |
| **SUB-SPECIALTY** |  |  |  |  |
| COLORECTAL/ILEAL | 1.577284 | 1.127347 | 1.399 | 0.16178 |
| ENDOCRINE | 0.61217 | 1.206551 | 0.507 | 0.61189 |
| GYNAECOLOGY | 0.61492 | 1.208365 | 0.509 | 0.61083 |
| LIVER/HPB | 2.647993 | 1.186405 | 2.232 | **0.02562** |
| OTHER | 2.30876 | 1.176045 | 1.963 | **0.04963** |
| THORACIC | 1.420276 | 1.218371 | 1.166 | 0.24373 |
| UGI/BARIATRICS | 0.706493 | 1.214289 | 0.582 | 0.56069 |
| UROLOGY | 2.223425 | 1.18913 | 1.87 | 0.06151 |
| VASCULAR | 1.408526 | 1.290641 | 1.091 | 0.27512 |
| **SURGICAL APPROACH** |  |  |  |  |
| ENDOSCOPIC | 0.043671 | 2.07967 | 0.021 | 0.98325 |
| LAPAROSCOPY/THORACOSCOPY | -0.12623 | 1.898329 | -0.066 | 0.94698 |
| OPEN | 0.222519 | 1.914176 | 0.116 | 0.90746 |
| ROBOTICS | -0.11729 | 1.860373 | -0.063 | 0.94973 |
| **CONVERSION (YES)** | 0.742092 | 1.753819 | 0.423 | 0.6722 |
| **SINGLE USE DISPOSABLE DEVICES (NUMBER)** | 0.035939 | 0.045834 | 0.784 | 0.43297 |
| **PROCEDURE (MINOR)** | 0.789364 | 0.518939 | 1.521 | 0.12823 |
| **PROCEDURE RE-ALLOCATED/UNPLANNED (YES)** | -0.42049 | 0.578933 | -0.726 | 0.46764 |
| **MULTI-ORGAN OR CONCOMITANT PROCEDURES (YES)** | 0.765501 | 0.581679 | 1.316 | 0.18817 |
| **PROCEDURE TIME (MINUTES)** | 0.016887 | 0.011206 | 1.507 | 0.13182 |
| **TIME OR OCCUPATION (MINUTES)** | -0.00996 | 0.010209 | -0.975 | 0.32934 |
| **DISCARDED FLUIDS (ML)** | -0.00029 | 0.000178 | -1.611 | 0.10718 |
| **BLOOD LOSS (ML)** | 0.001618 | 0.001029 | 1.572 | 0.11588 |
| **SURGEONS (NUMBER)** | 0.32332 | 0.234744 | 1.377 | 0.16841 |
| **NURSES (NUMBER)** | 0.530872 | 0.260241 | 2.04 | **0.04136** |

**^HPB:^** ^Hepatobiliary-Pancreatic;^ **^UGI:^** ^Upper Gastrointestinal^

**Supplementary Table 9. Correlations among variables**

**
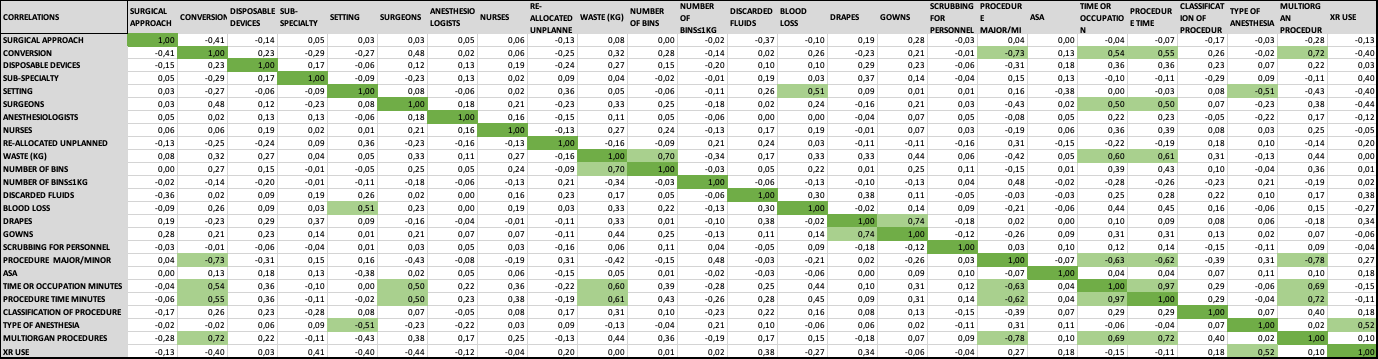
**

**Supplementary Table 10. Factor analysis**

| **VARIABLE** | **MR1** | **MR2** | **MR3** | **MR4** | **MR5** | **COMPLEXITY** | **UNIQUENESS** |
| --- | --- | --- | --- | --- | --- | --- | --- |
| **PROCEDURE TIME (MINUTES)** | 0.94 | \| | \| | \| | \| | 1.01 | 0.11 |
| **TIME OR OCCUPATION (MINUTES)** | 0.91 | \| | \| | \| | \| | 1.02 | 0.15 |
| WASTE (Kg) | 0.72 | \| | \| | \| | \| | 1.26 | 0.38 |
| NUMBER OF BINS | 0.57 | \| | \| | \| | \| | 1.25 | 0.69 |
| SURGEONS (NUMBER) | 0.53 | \| | \| | \| | \| | 1.55 | 0.63 |
| BLOOD LOSS (ML) | 0.51 | \| | \| | \| | \| | 1.51 | 0.73 |
| NURSES (NUMBER) | 0.46 | \| | \| | \| | \| | 1.35 | 0.8 |
| MULTI-ORGANS OR CONCOMITANT PROCEDURES | 0.39 | \| | \| | \| | \| | 2.01 | 0.73 |
| PROCEDURE (MAJOR/MINOR) | -0.32 | \| | \| | \| | \| | 2.56 | 0.71 |
| ANESTHESIOLOGISTS NUMBER) | 0.26 | \| | \| | \| | \| | 3.49 | 0.87 |
| NUMBER OF BINS WEIGHT<1KG | -0.25 | \| | \| | \| | \| | 2.67 | 0.83 |
| DRAPES | \| | 0.81 | \| | \| | \| | 1.04 | 0.32 |
| GOWNS | \| | 0.57 | \| | \| | \| | 1.31 | 0.6 |
| SCRUBBING FOR PERSONNEL | \| | -0.24 | \| | \| | \| | 2.36 | 0.94 |
| SURGICAL APPROACH | \| | \| | -0.84 | \| | \| | 1.03 | 0.29 |
| CONVERSION | \| | \| | 0.46 | \| | \| | 1.84 | 0.67 |
| DISCARDED FLUIDS | \| | \| | 0.34 | \| | \| | 4.13 | 0.62 |
| XR USE | \| | \| | 0.22 | \| | \| | 3.88 | 0.86 |
| SUB SPECIALTY | \| | \| | \| | 0.73 | \| | 1.01 | 0.44 |
| CLASSIFICATION OF THE PROCEDURE | \| | \| | \| | -0.31 | \| | 3.3 | 0.78 |
| SETTING | \| | \| | \| | \| | 0.34 | 1.42 | 0.88 |
| SINGLE USE DISPOSABLE DEVICES (NUMBER) | \| | \| | \| | \| | -0.31 | 4.23 | 0.69 |
| ASA SCORE | \| | \| | \| | \| | -0.2 | 2.23 | 0.93 |
| TYPE OF ANESTHESIA | \| | \| | \| | \| | 0.12 | 3.4 | 0.96 |

**Supplementary Table 11. Structural equation model (SEM)**

| **REGRESSIONS* OUTCOME: WASTE (KG)** |  |  |  |  |
| --- | --- | --- | --- | --- |
| SURGICAL SUB-SPECIALTY-WASTE | 1.122 | 0.339 | 3.309 | **0.001** |
| CLASSIFICATION OF THE PROCEDURE -WASTE | 0.738 | 0.3 | 2.457 | **0.014** |
| NURSES- WASTE | 0.84 | 0.231 | 3.643 | **0** |
| SURGEONS-WASTE | 0.689 | 0.162 | 4.262 | **0** |
| PROCEDURE MAJOR/MINOR-WASTE | -0.674 | 0.351 | -1.918 | 0.055 |
| MULTI-ORGANS OR CONCOMITANT PROCEDURES -WASTE | 0.803 | 0.375 | 2.14 | **0.032** |
| XR USE - WASTE | 0.036 | 0.509 | 0.071 | 0.944 |
| SETTING - WASTE | 0.394 | 0.935 | 0.421 | 0.674 |
| TYPE OF ANESTHESIA-WASTE | -0.212 | 0.4 | -0.529 | 0.597 |
| DRAPES - WASTE | 1.762 | 0.303 | 5.821 | **0** |
| GOWNS - WASTE | 0.949 | 0.365 | 2.604 | **0.009** |
| **REGRESSIONS OUTCOME: NUMBER OF BINS** |  |  |  |  |
| GOWNS | 0.338 | 0.089 | 3.794 | 0 |
| SURGICAL APPROACH | -0.137 | 0.056 | -2.457 | **0.014** |
| CLASSIFICATION OF THE PROCEDURE | -0.012 | 0.08 | -0.153 | 0.878 |
| SUB-SPECIALTY | 0.35 | 0.09 | 3.909 | 0 |
| SURGEONS | 0.094 | 0.044 | 2.156 | **0.031** |
| NURSES | 0.231 | 0.061 | 3.797 | 0 |
| PROCEDURE MAJOR/MINOR | 0.058 | 0.092 | 0.635 | 0.525 |
| **COVARIANCES** |  |  |  |  |
| SETTING ~~ TYPE OF ANESTHESIA | -0.001 | 0.001 | -0.45 | 0.653 |
| XR-USE ~~ TYPE OF ANESTHESIA | 0.014 | 0.003 | 4.994 | 0 |
| DRAPES ~~ GOWNS | 0.072 | 0.009 | 8.356 | 0 |
| PROCEDURE MAJOR/MINOR ~~ CONVERSION | -0.008 | 0.004 | -1.933 | 0.053 |
| MULTI-ORGANS OR CONCOMITANT PROCEDURES ~~ CONVERSION | 0.016 | 0.003 | 5.437 | 0 |
| PROCEDURE MAJOR/MINOR ~~ MULTI-ORGANS OR CONCOMITANT PROCEDURES | -0.014 | 0.005 | -2.542 | 0.011 |
| WASTE (KG) ~~ NUMBER OF BINS | 1.223 | 0.112 | 10.888 | 0 |
| SUB-SPECIALTY ~~ CLASSIFICATION | 0.076 | 0.012 | 6.478 | 0 |
| SUB-SPECIALTY ~~ NURSES | 0.032 | 0.014 | 2.327 | 0.02 |
| SUB-SPECIALTY ~~ SURGEONS | 0.059 | 0.02 | 2.98 | 0.003 |
| SUB-SPECIALTY ~~ SURGICAL-APPROACH | 0.031 | 0.011 | 2.699 | 0.007 |
| CLASSIFICATION OF THE PROCEDURE ~~ NURSES | 0.017 | 0.015 | 1.099 | 0.272 |
| CLASSIFICATION OF THE PROCEDURE ~~ SURGEONS | 0.027 | 0.022 | 1.21 | 0.226 |
| CLASSIFICATION OF THE PROCEDURE ~~ SURGICAL APPROACH | 0.046 | 0.013 | 3.638 | 0 |
| NURSES ~~ SURGEONS | 0.11 | 0.028 | 3.946 | 0 |
| NURSES ~~ SURGICAL APPROACH | 0.016 | 0.016 | 1.031 | 0.303 |
| SURGEONS ~~SURGICAL APPROACH | -0.114 | 0.023 | -4.918 | 0 |
| **VARIANCES** |  |  |  |  |
| WASTE (KG) | 6.682 | 0.495 | 13.491 | 0 |
| NUMBER OF BINS | 0.463 | 0.034 | 13.491 | 0 |
| PROCEDURE MAJOR/MINOR | 0.15 | 0.011 | 13.491 | 0 |
| MULTI-ORGANS OR CONCOMITANT PROCEDURES | 0.069 | 0.005 | 13.491 | 0 |
| XR USE | 0.04 | 0.003 | 13.491 | 0 |
| SETTING | 0.011 | 0.001 | 13.491 | 0 |
| TYPE OF ANESTHESIA | 0.064 | 0.005 | 13.491 | 0 |
| DRAPES | 0.136 | 0.01 | 13.491 | 0 |
| GOWNS | 0.16 | 0.012 | 13.491 | 0 |
| CONVERSION | 0.042 | 0.003 | 13.491 | 0 |
| SUB-SPECIALTY | 0.189 | 0.014 | 13.491 | 0 |
| CLASSIFICATION OF THE PROCEDURE | 0.234 | 0.017 | 13.491 | 0 |
| NURSES | 0.365 | 0.027 | 13.491 | 0 |
| SURGEONS | 0.748 | 0.055 | 13.491 | 0 |
| SURGICAL APPROACH | 0.244 | 0.018 | 13.491 | 0 |
| **DEFINED PARAMETERS:** |  |  |  |  |
| DIRECT EFFECT - WASTE | 0.001 | 0.019 | 0.067 | 0.947 |

*REGRESSION (CORRECTED FOR FACTOR ANALYSIS)

**Supplementary Table 12. SEM Performance**

| TEST STATISTIC | 269.344 |
| --- | --- |
| DEGREES OF FREEDOM | 70 |
| P-VALUE (CHI-SQUARE) | 0.000 |

**Supplementary Table 13. Confirmatory Factor Analysis of SEM**

| **PARAMETER** | **CUT-OFF FOR SIGNIFICANCE** | **SEM MODEL RESULT** |
| --- | --- | --- |
| COMPARATIVE FIX INDEX (CFI) | ≥0.90 | 0.741 |
| ROOT MEAN SQUARE RESIDUAL RMSE (RMSE-CI LOWER-RMSE-CI UPPER) | <0.05 | 0.089 (0.078 - 0.100) |
| RMSE P-VALUE | <0.05 | 0.000 |
| STANDARDIZED ROOT MEAN SQUARE RESIDUAL (SRMR) | <0.08 | 0.092 |
| TUCKER-LEWIS INDEX (TLI) | >0.90 | 0.648 |

**Supplementary Figure 1. Map of the Institution**

**Supplementary Figure 2. Data captured in the audit**

**A.** Data not captured; **B.** Data captured

**Supplementary Figure 3. Map of the direct and indirect effects**

**A.1.** Indirect effect of collinear variables and their inter-relations (conversion-major/minor procedure/multi-organ procedures); **A.2**. Indirect effect of collinear variables and their inter-relations (setting - XR use - type of anesthesia - blood loss); **A.3**. Indirect effect of collinear variables (drapes and gowns); **B.** Direct effects of non-collinear variables; **C.** Outcomes of interest.

**Supplementary Data Interpretation and Limitations**

This audit provided an exhaustive cross-sectional evaluation of surgical practices concerning OR waste, identifying several issues that present opportunities for improvement in waste reduction. While a few of these issues offer limited scope for improvement, all can be reconsidered to enhance waste management efficiency. Some problems, such as multiple organ resections, contamination during procedures, and lengthy surgical times, are significantly linked to waste output but are challenging to address. In contrast, other areas, particularly CRC and HPB surgeries or emergency and re-allocated procedures, could be prioritized for targeted interventions.

As an example, the re-allocation of procedures increases the OR performance with efficient management of surgical lists' allocations; although inevitable, it correlated with an increased waste output. This could be somehow expected, since an OR prepared for a day of urology procedures, could -for example- require a readjustment of materials if it ends with an HPB or UGI resection.

Additionally, the study revealed significant levels of waste output correlated with practices that could be easily revised, like the use of non-woven fabric drapes and gowns and the increased number of disposable devices.

These findings are consistent with patterns observed in other studies, which highlight the role of disposables in waste generation. More than 30 years ago, it was already documented that using reusable linens could reduce the weight of surgical waste by more than 70%.^10^ However, the change in practice could involve also a re-thinking: if nearly 80% of the procedures tracked in this audit were classified as major procedures requiring fully draped fields and glowed surgeons, minor surgeries could be suitable for different draping policies (i.e. single fenestrated drape plus sterile glows for surgeons), and this simple measure was documented effective in reducing surgical waste.^11^ On the other hand, the excess use of single-use devices, has been largely correlated to surgical waste: a past study in this field reduced the number of laparoscopic appendectomy instruments by more than 50%, downsizing emissions and costs for each procedure,^12^ demonstrating also the feasibility of this implementation. However, the impact of laparoscopy on the carbon footprint was not documented by others. ^13^ This issue, in particular, should consider multiple dimensions of the surgical practice, for example, the time of procedure (single-use energy devices reduce operative time) and time of hospitalization (reduced by the use of mini-invasive surgeries).

The greatest impact in this audit was related to conversion (as expected the shift from mini-invasiveness to open surgery was documented as correlated to an increased median weight of surgical waste and an increased number of cardboard bins) and the use of robotic platforms (see Supplementary Methods). Of note, in the present study, we did not consider robotic arms as disposable, and to this end, robotics could be perceived as the mini-invasive approach providing more reusable types of equipment.

The use of reusable rather than single-use devices could be a game-changer in this field: however, even if reusable laparoscopic instruments were documented as safe and effective in randomized studies,^14^ their uptake in the general market is still limited.

On the other hand, the results of the multivariable models pointed more to surgical sub-specialty and personnel involved in each procedure: as a university hospital, however, the number of surgeons, nurses, and anesthesiologists is related to teaching activities to trainees and fellows and it should be taken into consideration.

However, and despite the number of correlations identified, we could not recognize any causal-effect relation. Indeed, while using a structural equation modeling framework offers insights into inter-variable correlations,^7^ causal relationships remained speculative due to potential confounding factors not captured in the model.

Finally, it was documented that the simple intervention of reducing the number of cardboard bins in the OR could impact the emissions related to transportation. Indeed, cardboard bins could contain up to 60L of waste, but their distribution could be implemented easily by rethinking the unnecessary, evaluating, and re-assessing all elements, practices, or resources that could be outdated or inefficient in waste management. This process aims to critically analyze what is truly essential and to question whether other habits or procedures can be eliminated or improved to enhance sustainability. Strikingly, about 7% of the cardboard bins in the ORs had a weight less or equal to 1 kg (resulting in a net weight of less than 300 gr). This is of paramount importance when considering the emissions estimated by this audit. To have a rough estimate, the emissions generated during the audit corresponded to driving a vehicle for approximately 1.5 months (assuming continuous driving) or 6.71 round-trip flights from New York to Los Angeles. However, we should consider that these figures may vary depending on the type of engine, the size of the vehicles, and the number of passengers.

On the other hand, the use of recyclable bins was not documented as effective: this is in contrast with what was reported by others, even if the experience was limited to metal items.^15^

A key strength of this study is its comprehensive approach, incorporating a wide array of surgical specialties within the largest research university hospital in Italy.

Despite the study's robustness, several limitations exist: firstly, we have to acknowledge that we investigated just a segment of the carbon footprint generated by the OR, namely: the waste output.

Waste is nevertheless the most commonly reported outcome of interest, being acknowledged as the metric reported by 57-62% of the literature in this field.^16-17^

Secondly, although we reported on more than 360 procedures and modeled the emissions on the annual estimates of a high-volume institution, this remains a single-center audit, like 56.5% of the literature in this field.^17^ It should be noted that, as a single-center experience, the strength relies on the homogeneous practices established inside the Institution, with a minimal impact in terms of variation in hospital management. Moreover, this study presents a multi-specialty perspective that adds a novel understanding of institutional waste patterns, allowing for a broader interpretation of ecological impacts within the surgical context.

Regarding the study's duration, though adequate for auditing purposes, it reflects a snapshot that may not capture seasonal variations in surgical activities. Additionally, the exclusion of certain procedures (e.g., cardiac and neurosurgery) limits the applicability of findings to these specialties.

Regarding anesthesiology, it should be noted that the vast majority of patients during the audit received mixed-balanced anesthesia (nearly 90%), while only a minority were managed using other approaches. This practice is indeed a limitation for case mix; however, it is the standard anesthesiology practice in our institution, which follows ERAS recommendations whenever possible. Additionally, the management of piped gas is well-controlled, as it is administered using a closed circuit with minimal dispersion and is regularly monitored by the Health Department.

Also, other aspects were not considered including the packing and unpacking modalities of surgical instruments and trays, the impact of sterilizations for surgical instruments, energy and water consumption, reduction of equipment and downsizing of surgical trays, recycling of disposables, implementation in waste segregation and recycling. Each of these elements could result in a reduction of the carbon footprint.

Indubitably, changing waste management in the OR requires time, personnel education, clear policies, and the overcoming of cultural barriers and personal habits, but it is also a rewarding opportunity in which hospital management should invest.

Finally, the estimation of the emissions was calculated based on the most common trucks used in Europe. We acknowledge that these calculations may change based on the vehicles' weight and engine, as well as changes in the market and EU policies (for example, hybrid engines and other types of transportation). This study offers valuable insights into OR waste production, presenting implementable models for reducing emissions. As surgical activities continue to increase globally, our findings underscore the critical need for healthcare systems to integrate sustainable practices into OR management. With further research, particularly across diverse institutions and longer timeframes, healthcare providers can better tailor interventions to meet ecological targets and enhance sustainability in surgical care.

**Supplementary References**

**1.** Bahl V, Hu HM, Henke PK, Wakefield TW, Campbell DA Jr, Caprini JA. A validation study of a retrospective venous thromboembolism risk scoring method. *Ann Surg* 2010;**251:**344-50.

**2.** Chen M, Zhou R, Du C, Meng F, Wang Y, Wu L, Wang F, Xu Y, Yang X. The carbon footprints of home and in-center peritoneal dialysis in China. *Int Urol Nephrol* 2017;**49:**337-343.

**3.** Connor A, Lillywhite R, Cooke MW. The carbon footprint of a renal service in the United Kingdom. *QJM* 2010;**103:**965-75.

**4.** Duane B, Hyland J, Rowan JS, Archibald B. Taking a bite out of Scotland's dental carbon emissions in the transition to a low carbon future. *Public Health* 2012;**126:**770-7.

**5.** Lim AE, Perkins A, Agar JW. The carbon footprint of an Australian satellite haemodialysis unit. *Aust Health Rev* 2013;**37:**369-74.

**6.** Mulholland E, Ragon PL, Rodríguez F. CO2 emissions from trucks in the European Union: An analysis of the 2020 reporting period. WORKING PAPER 2023-14. © 2023 INTERNATIONAL COUNCIL ON CLEAN TRANSPORTATION. Available from: https://theicct.org/wp-content/uploads/2023/07/hdv-co2-emissions-eu-2020-reporting-2-jul23.pdf.

**7.** Lorenzon L, Caccialanza R, Casalone V, Santoro G, Delrio P, Izzo F, Tonello M, Mele MC, Pozzo C, Pedrazzoli P, Pietrabissa A, Fenu P, Mellano A, Fenocchio E, Avallone A, Bergamo F, Nardi MT, Persiani R, Biondi A, Tirelli F, Agnes A, Ferraris R, Quarà V, Milanesio M, Ribero D, Rinaldi M, D'Elia P, Rho M, Cenzi C, D'Ugo D. The impact of preoperative nutritional screening, ERAS protocol, and mini-invasive surgery in surgical oncology: A multi-institutional SEM analysis of patients with digestive cancer. *Front Nutr* 2023;**10:**1041153.

**8.** Agenas: accessed September 13th 2024. Available from: https://pne.agenas.it/ospedaliera/strutture/12090501.

**9.** Rizan C, Lillywhite R, Reed M, Bhutta MF. Minimising carbon and financial costs of steam sterilisation and packaging of reusable surgical instruments. *Br J Surg* 2022;**109:**200–210.

**10.** Tieszen ME, Gruenberg JC. A quantitative, qualitative, and critical assessment of surgical waste. Surgeons venture through the trash can. *JAMA* 1992;**267:**2765-8.

**11.** Sinha V, Zolfaghari Y, Vijayan R. The great cover up: Environmental toll of excessive surgical draping in minimally invasive surgery. *J Plast Reconstr Aesthet Surg* 2023;**82:**209–210.

**12.** Boag K, Ho T, Quyn A, Peckham-Cooper A. A sustainable appendicectomy. *Br J Surg* 2022;**109:**75. <https://doi.org/10.1093/bjs/znac248.144>

**13.** Gilliam AD, Davidson B, Guest J. The carbon footprint of laparoscopic surgery: should we offset? *Surg Endosc Other Interv Tech* 2008;**22:**573.

**14.** Colak T, Ersoz G, Akca T, Kanik A, Aydin S. Efficacy and safety of reuse of disposable laparoscopic instruments in laparoscopic cholecystectomy: a prospective randomized study. *Surg Endosc* 2004;**18:**727–731.

**15.** Sadler A, Wilde P, Raju P, Rodney G. A trial of metal recycling in healthcare waste management. *Anaesthesia* 2017;**72:**10–88.

**16.** Balch JA, Krebs JR, Filiberto AC, Montgomery WG, Berkow LC, Upchurch GR Jr, Loftus TJ. Methods and evaluation metrics for reducing material waste in the operating room: a scoping review. *Surgery* 2023;**174:**252-258.

**17.** Huo B, Eussen MMM, Marconi S, Johnson SM, Francis N, Oslock WM, Marfo N, Potapov O, Bello RJ, Lim RB, Vandeberg J, Hall RP, EdM AAMD, Sanchez-Casalongue M, Alimi YR, Pietrabissa A, Arezzo A, Frountzas M, Bellato V, Barach P, Rems M, Nijihawan S, Sathe TS, Miller B, Samreen S, Chung J, Bouvy ND, Sylla P. Scoping review for the SAGES EAES joint collaborative on sustainability in surgical practice. *Surg Endosc* 2024 Aug 22. doi: 10.1007/s00464-024-11141-x. Epub ahead of print. PMID: 39174709.
